# HOME Plus: Program design and implementation of a family-focused, community-based intervention to promote the frequency and healthfulness of family meals, reduce children’s sedentary behavior, and prevent obesity

**DOI:** 10.1186/s12966-015-0211-7

**Published:** 2015-04-29

**Authors:** Colleen Flattum, Michelle Draxten, Melissa Horning, Jayne A Fulkerson, Dianne Neumark-Sztainer, Ann Garwick, Martha Y Kubik, Mary Story

**Affiliations:** School of Nursing, University of Minnesota, 5-160 Weaver-Densford Hall, 308 Harvard St. SE, Minneapolis, MN 55455 USA; Department of Family Medicine, University of Minnesota, Minneapolis, MN USA; Department of Epidemiology and Community Health, School of Public Health, University of Minnesota, Minneapolis, MN USA; Community and Family Medicine and Global Health, Duke University, Durham, NC USA

**Keywords:** Family meals, Dietary quality, Behavioral intervention, Motivational Interviewing, Obesity prevention

## Abstract

**Background:**

Involvement in meal preparation and eating meals with one’s family are associated with better dietary quality and healthy body weight for youth. Given the poor dietary quality of many youth, potential benefits of family meals for better nutritional intake and great variation in family meals, development and evaluation of interventions aimed at improving and increasing family meals are needed. This paper presents the design of key intervention components and process evaluation of a community-based program (Healthy Home Offerings via the Mealtime Environment (HOME) Plus) to prevent obesity.

**Methods:**

The HOME Plus intervention was part of a two-arm (intervention versus attention-only control) randomized-controlled trial. Ten monthly, two-hour sessions and five motivational/goal-setting telephone calls to promote healthy eating and increasing family meals were delivered in community-based settings in the Minneapolis/St. Paul, MN metropolitan area. The present study included 81 families (8-12 year old children and their parents) in the intervention condition. Process surveys were administered at the end of each intervention session and at a home visit after the intervention period. Chi-squares and t-tests were used for process survey analysis.

**Results:**

The HOME Plus program was successfully implemented and families were highly satisfied. Parents and children reported that the most enjoyable component was cooking with their families, learning how to eat more healthfully, and trying new recipes/foods and cooking tips. Average session attendance across the ten months was high for families (68%) and more than half completed their home activities.

**Conclusions:**

Findings support the value of a community-based, family-focused intervention program to promote family meals, limit screen time, and prevent obesity.

**Trial registration:**

NCT01538615

## Background

Diet quality of children and adolescents has long been a concern for health professionals and researchers. Despite recommendations of the 2010 Dietary Guidelines for Americans, youth typically have inadequate intakes of fruits, vegetables and whole grains and excessive intake of added sugar and fat [1-3]. Involvement in meal preparation and eating meals with one’s family are associated with better dietary quality and healthy body weight for youth [4-8], making the promotion of family meals a possible nutrition- and weight-related health promotion strategy [7,9-14].

Interventions that strive to teach youth about the importance of healthful eating have been conducted across communities with varying success [15-17]. Creative and innovative programs such as hands-on cooking classes and gardening programs for youth [18-20] continue to be designed and delivered to children and adolescents to promote healthful eating. Current research suggests that involving youth in food preparation is associated with a preference for healthy eating [13,21,22]. However, detailed information beyond general statements about promotion of food preparation involvement and frequency is typically unavailable.

Given the poor dietary intake of many youth [2,3], potential benefits of family meals for better nutritional intake [4,9,10,23] and great variation in family meals, it is crucial to develop interventions aimed at improving and increasing family meals and evaluate their potential impact on diet quality and obesity. However, little research has evaluated intervention programs from a family-focused, behavior change perspective. Therefore, this paper presents an overview of key components of the Healthy Home Offerings via the Mealtime Environment (HOME) Plus program, a family-focused program to promote family meal frequency and healthful meals and snacks among 8–12 year-old children and their families. The theoretical model, guiding principles, intervention session components, and important process evaluation components [24,25] such as intervention fidelity, delivery and receipt dosage (attendance), responsiveness and use (parent and child satisfaction and homework completion) and self-evaluation of change are discussed.

## Methods

### Study design and participants

The HOME Plus program is currently being evaluated in a randomized controlled trial with160 families (one target 8–12 year old child per family and the primary meal-preparing parent/guardian) with three data collection periods: baseline (2011, 2012), post-intervention (post-intervention) and follow-up (9 months post-intervention). After baseline assessment, families were randomized to an intervention group (n = 81) and attended 10-monthly group sessions (Oct 2011-Jul 2012 and Oct 2012-Jul 2013, respectively for cohorts 1 and 2) or an attention-only control group (n = 79) that received 10-monthly newsletters. A staggered cohort design was used to accommodate the capacity of community centers and staff within funding limits.

Families were recruited from community centers in six geographic locations of Minneapolis/St. Paul, Minnesota’s metropolitan area. Recruitment efforts were targeted to primary meal-preparing parents of 8–12 year old children to increase the likelihood of accurate reporting related to food preparation and making changes in the home food environment. Effective methods such as flyers and small group presentations used successfully in the pilot study were used for recruitment [18]. Community center staff assisted with recruitment and facilitated logistics during intervention sessions. Parent and child participants signed informed consent or assent forms, respectively, and completed assessments including psychosocial surveys, anthropometric measures, dietary recall interviews (child only) and home food environmental measures. All procedures were approved by the University of Minnesota’s Human Subjects Review Board. Study design, methods, eligibility and detailed data collection information is published elsewhere [26].

Children participating in the intervention were 8–12 years old (M = 10.5 years, SD = 1.5); 69% were white, 16% African American/Black and 15% mixed race/ethnicity; 46% were female; and 41% were overweight/obese (>85%BMI percentile). Most participating parents in the intervention were female (94%); 78% of parents were white, 15% African American and 7% mixed race/ethnicity. Many parents were college educated (70%) and 48% were working full-time. Because income level is dependent upon household size, receipt of economic assistance (free and/or reduced lunch for child at school and/or public assistance through food support/stamps, EBT, WIN, TANF, SSI or MFIP) was used to measure household economic status; almost half of parents (45%) reported receiving economic assistance. The parent average age was 41 years (SD = 8.0) and 46% were overweight/obese.

### Program description

A stepwise approach to designing and developing the HOME Plus intervention was used to maximize the program’s likely effect [27]. The formative steps included: 1) targeted behavior validation, (i.e., obesity prevention of 8–12 year old children); 2) targeted mediator validation (i.e., Social Cognitive Theory (SCT) (personal, behavioral, and environmental factors)); 3) intervention procedure validation, (i.e., skill development and education); and 4) pilot/feasibility of the intervention. Process evaluation was conducted throughout the intervention to assess fidelity, dosage, responsiveness and satisfaction.

HOME Plus was based on a family meal program (HOME) previously developed and pilot tested for feasibility and acceptability by our team in 2006–2008 [18], with the addition of a component to reduce sedentary behavior (mainly screen time). HOME Plus was guided by Social Cognitive Theory (SCT) and a socio-ecological framework [28-30]. As shown in Table 1, the intervention had three overarching goals associated with behavioral messages related to the planning, frequency and healthfulness of family meals and snacks.

**Table 1:** **HOME Plus goals and behavioral messages for intervention families**

**Goals**	**Behavioral Messages**
1. Plan healthy meals and snacks with your family more often	• Get kids involved with shopping at least three times a month
	• Plan and prepare healthy meals and snacks together at least three times a week
	• Plan family meals and snacks using portion size guidelines
	• Creatively involve kids in trying new fruits and vegetables
	• Make half your plate fruits and vegetables at meals
2. Have meals with your family at home more often	• Make regular family meals a priority
	• Enjoy your food, but don’t overeat
	• Sit together during meal time
	• Promote positive conversation at meal time
	• Eliminate electronics at meal time
3. Improve the healthfulness of the food available at home	• Increase the amount and variety of fruits and vegetables in the home
	• Make fruits and vegetables more visible and easily accessible in the home
	• Reduce the number of high fat and high sugar snacks in the home by at least half
	• Replace sugar-sweetened beverages with water
	• Rely less on highly processed foods in the home

Sessions incorporated concepts of SCT, such as increasing self-efficacy of both parents and children (e.g., through hands-on cooking activities designed to increase skills/confidence), increasing the outcome expectation of eating healthful food (e.g., by being given the opportunity to consume healthful foods created at the intervention) and enhancing parental skill development (e.g., parents learn and practice how to praise children for trying new foods, limit screen time at meals, and avoid mixed messages about food, activity and weight).

### Intervention delivery

Intervention messages were addressed in a participant guidebook, *Let’s Eat Together–Your Family’s Guide to HOME Plus*, given to each family and utilized throughout the sessions. The guidebook included session topics, strategies to help meet session goals, recipes and resources (e.g., list of local farmer’s markets).

Intervention sessions were delivered monthly to multiple family groups at community park and recreation centers in the Minneapolis area in the early evening to accommodate family schedules. All family members were encouraged to attend. Childcare for children (<8 years) and transportation were available, as needed, to enhance retention and adherence. Each session was offered twice a month at each location, to allow for scheduling flexibility. Five brief goal-setting telephone calls were conducted by lead facilitators with intervention parents over the 10-month intervention. Details of intervention components are described below.

### Intervention components

#### Family group sessions

Lead facilitators used a set curriculum for intervention delivery (see Table 2 for brief content summaries). Sessions consisted of nutrition education and hands-on skill development to provide parents and children with new knowledge and practical application. Each session included 1) introduction of a new topic and review of prior month’s topic and goals (family); 2) meal preparation (family); 3) taste testing a seasonal fruit/vegetable (separate parent and child groups); 4) small break-out groups with discussion and activity (separate parent and child groups); 5) eating a family meal (family); and 6) summary of session (family). Some details of a typical session are described below.

**Table 2:** **HOME Plus Session Topics with Parent and Child Ratings of Each Session**

**Session**	**Topics**	**Mean rating of session**
		**(1 = didn’t like, 5 = loved it)**
1-Let’s get started	Best family meal ever	Parent = 4.4 Child = 4.2
	Wash, chop, slice and safety…kitchen basics	
2-Ready, Set, Goal	Goal setting…breaking it into bite-size pieces	Parent = 4.4 Child = 4.1
	Let’s give them something to talk about–conversation starters	
	Recipe revolution–common abbreviations	
3-Thinking outside the box	Switch it up: meal planning makeovers	Parent = 4.4 Child = 4.3
	Go! Slow! Whoa!	
	Successful recipes = accurate measures	
4- What’s for dinner 2night?	Cook today, eat tomorrow–or freeze for another day	Parent = 4.4 Child = 4.0
	READ it before you EAT it	
	A dash of this, a pinch of that–measuring ingredients	
5-Too much? Not enough?	Portion distortion– helpings, portions and servings	Parent = 4.6 Child = 4.3
	Are you hungry? Full? Listening to your body’s cues	
	Get creative-colorful, fresh and nutritious salads	
6-Keep it under wraps	Fast, fun and full of acceptance–ideas for picky eaters	Parent = 4.4 Child = 4.1
	Making sense of advertising	
	Wrap it up-Quick and easy meals	
7-Balance, balance, keep the balance	Healthy snacks-beyond apples and oranges	Parent = 4.6 Child = 4.3
	The race is on…choosing healthy snacks	
	Peel! Chop! Fruits!	
8-Less sugar and fat–a sweet deal	Sip smarter–the bottom line on sugary drinks	Parent = 4.6 Child = 4.4
	Which snack or beverage? Check the facts!	
	Peel! Chop! Vegetables!	
9-AGREENable meals and snacks	Why your choices matter	Parent = 4.5 Child = 4.3
	Celebrate seasons–picking produce that’s fresh & less expensive	
10-The future is bright…planning ahead	The Celebrity Chef is…you!	Parent = 4.7 Child = 4.5
	Kids can do it…families can do it	

Upon session arrival, each family selected one of four featured recipes to prepare (meat entree, vegetarian entree, salad, or fruit-based dessert). Parents and children were introduced to new recipes, developed basic knife skills, and practiced reading a recipe and measuring ingredients. These skills were targeted to promote meal planning and preparation self-efficacy. Recipes were selected based on the Dietary Guidelines for Americans (i.e., recipes contained 30% or less of calories from fat/serving and promoted fruits/vegetables). For simplicity, recipes had few overall ingredients and emphasized highly-available and low-cost ingredients.

All participating family members sampled a seasonal fruit/vegetable in a *Taster’s Choice* activity to increase their exposure to a variety of fruits/vegetables. Fruits/vegetables selected for this activity were those that children rated during baseline data collection as ones they had “not tried” or “did not like.” Families were encouraged to try, on their own, the fruit/vegetable of the month before the next session as their *Take HOME activity*, which targeted the behavioral goal of increasing the number of fruits/vegetables available in the home and served at family meals and snacks. To encourage session attendance and completion of *Take HOME activities*, families received entries for a final session drawing for a personal home visit by a local chef.

#### Small group discussions and activities

Parent session activities focused on reducing barriers and strategies for behavior change related to program messages. For example, parents discussed mealtime stress, ways to increase the frequency and healthfulness of family meals, and strategies to increase healthful snacks at home through role-play and case scenarios. Children’s group topics paralleled those of the parent but were more game-like to educate them in a developmentally-appropriate and engaging manner.

Sessions concluded with family-style meals where families tried the foods made by the group. A pre-portioned plate was on display at every meal to demonstrate appropriate serving sizes. All participants were encouraged to try at least a sample of each food. Following dinner, parents and children completed session evaluations and selected family-level goals, i.e., a goal that all members of the family agreed they could work toward, for the next month (for example, increase the amount of fruits and vegetables as snacks). Families unable to attend a session received a telephone call from their facilitator who recapped the session and mailed them pertinent handouts.

#### Parent goal-setting telephone calls

Five brief (~20 minute) tailored goal-setting telephone calls were conducted by lead facilitators, who were trained in Motivational Interviewing (MI), with parents over the 10-month intervention. Often during the calls, parents selected new goals to complement the family-selected goal at sessions and tended to be focused on parental strategies for feeding picky eaters or eliminating junk food from the home. Parents had the option of working on the same goal throughout the intervention or choosing a new goal at any point. Each call followed a counseling protocol based on MI principles, including a participant-focused, collaborative, decision-making approach, giving nonjudgmental feedback, allowing for resistance, and encouraging the participant to make a case for change [31,32]. The facilitators relied on open-ended questions and reflections to bring about the participant’s motivation and desire for change. Intervention staff held weekly case management meetings to discuss and address problem areas.

### Program cost

Cost estimates were broken down to include training of intervention personnel, one-time program materials and costs associated with intervention delivery by family. Costs per family were as follows: One-time cost of $20 for personnel training (first aid and food safety training, study t-shirt and chef hat (as uniform)), one-time cost of $49 for program materials for participants at the beginning of the program (guidebook, recipe book, chef hat and canvas bag), and $44 per session for intervention delivery (staff time ($27 per family), food ($8 per family), small incentives ($3 per family), room rental ($6 per family)). In addition, childcare cost $20 per session for up to 6 kids and transportation cost $12.50 per session for families (n = 6) requiring cab or bus transportation. It is important to note that at least three college students volunteered to assist with session logistics at each session as well.

### Intervention process evaluation

#### Fidelity of program delivery

All intervention staff members were trained to study protocols and food safety practices; lead staff was also trained in basic first aid. Group sessions were facilitated by Registered Dietitians and a Registered Nurse. Lead staff that conducted the goal-setting phone calls were trained in MI prior to program start up. The program assistant supervised university-level student volunteers (usually 3 per session) in setting-up cooking stations. All team members assisted families during meal preparation and service.

Observations of session curriculum delivery were regularly conducted to monitor and enhance program fidelity [24,27,33]. Session observations were conducted at months 3, 6 and 9 by trained university-level students using a standardized checklist. The principal investigator monitored the checklists and reviewed them with staff.

#### Participant receipt dosage and responsiveness/use

Session attendance and goal-setting telephone call completion were the measures of program dosage. Study staff documented attendance of all family members at sessions and all telephone call attempts and completions. Homework completion of the *Take HOME activity* measured participant responsiveness/use. Additionally, we assessed if participants used their *Family Guidebook* and/or if they made session recipes at home. Lastly, parents and children self-evaluated any behavioral changes they attributed to HOME Plus.

#### Participant satisfaction

Parents and children completed satisfaction measures of the overall HOME Plus program. They were also asked if they would recommend the program to friends/family and to provide reasons for participation in the program.

## Results

### Fidelity of program dose and delivery

Staff training was standardized using protocols and manuals. Based on fidelity monitoring, 90% of sessions were delivered as intended. The main deviation was program start time; delays occurred because some families did not arrive on time.

### Participant receipt dosage and responsiveness/use

As shown in Table 3, 68% of families attended at least 7 of 10 sessions (high dosage). Motivational/goal-setting call completion was high (87% average) with 84% of families completing at least 4 of 5 calls (high dosage). In terms of overall intervention delivery, 31% completed all sessions *and* all calls; 54% completed some sessions *and* some calls (≥5 sessions and ≥3 calls); 11% completed some sessions *or* some calls (≥1sessions and ≥1 calls) and 4% completed no session or calls.

**Table 3:** **Participant Rates for HOME Plus Group Sessions and Goal-Setting Phone Calls (n = 81 families)**

**Session**	**Attendance**	**Call**	**Completion**
	**N families (%)**		**N families (%)**
1	64 (79%)	1	76 (94%)
2	61 (75%)	2	73 (91%)
3	54 (67%)	3	70 (88%)
4	56 (70%)	4	64 (80%)
5	50 (63%	5	64 (80%)
6	50 (63%)		
7	55 (69%)		
8	50 (63%)		
9	46 (58%)		
10	57 (70%)		
**Average**	54 (68%)		69 (87%)

Given the small numbers in the low dosage categories of both sessions and calls, we were only able to assess for dosage differences by family demographic characteristics using a dichotomous low/high dosage of sessions (i.e., <7 vs 7+) and calls (i.e., <4 vs 4+). Parents attending a high dose of sessions were significantly more likely to identify as white (80%) compared to those identifying as black/mixed race/other (50%; χ^2^ (1) = 5.35, p = .02); similar findings were found by child race (data not shown). Because household income level does not account for family size, receipt of economic assistance was used to measure family economic status; families not receiving economic assistance (86%) were more likely to have high session dosage compared to families receiving economic assistance (57%; χ^2^(1) = 8.24, p < 0.01). Completion of a high dose of calls was not significantly associated with parent race, child race, or receipt of economic assistance. Both session and call dosage were not significantly associated with parent marital status or child age. Of families attending at least one session, 59% completed their *Take HOME Activity* and 75% brought additional family members to at least one session. Most (81%) families had made at least one HOME Plus recipe at home. Almost all parents (95%) reported motivational/goal-setting calls with staff were helpful reminders of program goals.

Program satisfaction was high; 98% of parents reported being “satisfied” or “very satisfied” with HOME Plus (Table 4). Parents reported cooking with their family/child, learning how to eat more healthfully, trying new recipes/foods and cooking tips as what they liked most about sessions (Table 4). Eighty-eight percent of children reported they would recommend HOME Plus to other children (Table 4). Over three-quarters of children reported HOME Plus increased their willingness to try new foods, eat more fruits and vegetables and eat healthier snacks (Table 4).

**Table 4:** **Parent and Child Participant-Reported HOME Plus Satisfaction**

**Question:**	**Parent N = 68**
*How satisfied were you with the HOME Plus program*	*66(98%)
*Because of the HOME Plus program…*	
I am more aware of portion sizes	*58(92%)
my child is more aware of his/her portion sizes	*54(86%)
my child is more open to trying new foods	*52(87%)
*What did you like most about participating in the HOME Plus program*	
Cooking with family/child	^10(32%)
Learning how to eat more healthfully	^6(19%)
Trying new recipes/foods and tips	^6(19%)
	**Child N = 71**
*Would you recommend the HOME Plus program to other kids?*	*59(88%)
*Because of the HOME Plus program…*	
I am willing to try new foods	*52(85%)
I eat more fruits and vegetables	*52(84%)
I eat healthier snacks	*51(82%)
*What components did you like best about the HOME Plus program?*	
taste-testing fruits	*53(79%)
cooking with my parent	*51(76%)
learning food preparation skills like chopping and measuring	*39(58%)

*satisfied/highly satisfied.

^open-ended.

## Discussion

This paper describes the intervention and process evaluation of the HOME Plus family meals intervention program. The study and program design were based on a strong body of literature demonstrating the importance of family meals for a variety of healthful outcomes among youth [34-37]. There is a dearth of intervention programming with a primary focus on family meals with entire families [38-41], and to the best of our knowledge, the HOME Plus intervention is the first family meals-focused intervention to be evaluated in a randomized controlled trial. The intervention design and detailed intervention protocol benefited from pilot work, a solid theoretical foundation and dedication of highly trained staff and student volunteers. The vibrant intervention was designed to enhance learning, promote behavior change for children and parents and engage all family members to facilitate family-level change. In combination with personalized motivational/goal-setting calls, facilitators provided the support necessary for family behavior change in multi-family groups in community settings. Findings from the current study indicate the high level of feasibility and acceptance of this community-based, family-focused intervention to promote family meals, reduce sedentary behavior and prevent obesity.

Child and parent self-evaluation of behavior change indicate cooking together, skill building and promoting family meals are valued activities. Parents provided particularly favorable ratings regarding the multiple, family-group format and group discussions at sessions, suggesting community-based delivery with groups is acceptable. Children indicated they were more willing to try new foods and eat more healthful foods because of HOME Plus, suggesting promotion of healthful eating through the family meal may be an important public health strategy. HOME Plus was highly regarded by both parents and children and filled a need for families who wanted to eat more family meals and promote healthful food in their home.

Parental engagement is a key contributor to effectiveness of family interventions [42,43]. Providing programming to address time constraints and needs of busy families through flexibility in session schedules; telephone calls to enhance session topics; ending each session on time; selecting quick, tasty and healthful recipes and intervention components best suited for the developmental age of participants were strategies contributing to the successful delivery of HOME Plus.

Strengths of HOME Plus include a standardized process for the design, application of quality assurance methods, successful engagement of the whole family and rigorous process evaluation. In addition, we have included detailed cost break down of study components which provides cost estimates of intervention delivery. This information can be used to guide budgets for similar research studies and/or programs within the community. However, program limitations also need to be considered, including variations within sites such as difficulty engaging families with variable needs and the children’s wide age range (8–12 years). However, every effort was made to accommodate family needs and deliver an intervention that was appropriate for the children’s developmental age. Finally, families who consented to participate in the study may have been more motivated to participate in a family meals-focused, healthful eating program compared to families who did not participate. Although, the study sample characteristics were representative of the county in which they were recruited, with the exception of being slightly more educated (which is not uncommon for health-related clinical trials), in-person session attendance was associated with less economic disadvantage and racial diversity. Since baseline family meal frequency was relatively high among intervention participants (M = 5.0, SD = 1.9), the program promoted frequent family meals but focused more on the healthfulness of meals at home (at baseline, 69% of families reported that fewer than half of their family meals were made at home). Despite these limitations, much has been learned about delivering a family-focused intervention and the program could be adapted for other communities, e.g., families who do not eat any family meals, and/or rural or low-income families, by conducting needs assessments, engaging communities to assess barriers, and using successful techniques like our MI calls.

## Conclusion

The success of the HOME Plus program delivery and participation illustrates offering programming to children and their parents focused on family meals in community settings are feasible and well-accepted. Programs are needed in which families feel comfortable planning and preparing meals, learning necessary skills and gaining knowledge to adopt healthful behavior changes. However, challenges are likely and need to be considered when promoting community-based, family-focused programs. Future family meal interventions, similar to HOME Plus, need to consider how to best implement the program and disseminate the program with less staff involvement. The HOME Plus intervention process findings suggest community-based, family-focused programs are achievable; this approach may represent an effective strategy to promote healthful family meals and prevent obesity among preadolescent youth.

## Abbreviation

SCT: Social Cognitive Theory
